# Editorial: Advances in the Bio- and Chemo-Catalytic Conversion of Biomass Components Into Biofuels and Value-Added Chemicals

**DOI:** 10.3389/fbioe.2021.769995

**Published:** 2021-10-18

**Authors:** Kai Yan, Chunbao Charles Xu, Guoqing Guan, Xu Wu

**Affiliations:** ^1^ Guangdong Provincial Key Laboratory of Environmental Pollution and Remediation Technology, School of Environmental Science and Engineering, Sun Yat-sen University, Guangzhou, China; ^2^ Department of Chemical and Biochemical Engineering, Western University, London, ON, Canada; ^3^ Institute of Regional Innovation, Hirosaki University, Aomori, Japan; ^4^ School of Chemistry and Chemical Engineering, Taiyuan University of Technology, Taiyuan, China

**Keywords:** homogeneous catalysis, heterogeneous catalysis, valorization, lignocellulose, biofuels, value-added chemicals, biocatalytic system

## Introduction

Biomass has been identified as a renewable alternative to fossil resources in producing promising transportation biofuels as well as highly valuable products (Huber et al., 2006; Alonso et al., 2012; Hu et al., 2021). Many crucial products, including fuels, fine chemicals and plastics, have been generated from the inedible portion of biomass resources and the fast increased need has sparked efforts on such transformation (Corma et al., 2007; Julis et al., 2010). The past several decades have witnessed a fast development of robust catalysts, various catalytic system, and catalytic pathways for converting lignocellulose into many useful products, making the valorization of renewable biomass into reality (Zhang et al., 2013; Zhang et al., 2020; Zhao et al., 2021). Catalyst development play an important role in achieving this goal. Over the last a few decades, different types of catalysts (e.g., metal nanoparticles, enzyme, acidic or basic candidates) have been frequently designed and investigated (Alonso et al., 2013; Nanda et al., 2016; Hu et al., 2019; Liu et al., 2021). This special issue concentrates on the pretreatment of biomass using enzymes, anaerobic digestion of naive source, various factors on the thermal pyrolysis, value-added chemicals synthesis and degradation.

## Biomass Pretreatment

The complex composition of naïve biomass makes it difficult to be utilized. Pretreatment is crucial for the further utilization. Saddler and coworkers reported the improved activity from the use of enzymes in tuning the hydrolysis of cellulose through introducing acid functions into the structure of lignin for the pretreatment. They found that the use of 16% Na_2_SO_3_ or 32% C_2_H_6_Na_4_O_12_ into the pulp would not induce clear delignification exhibiting the similar behavior to the proper dosage of HSO_3_^−^ and HCO_3_^−^ into the structure of lignin. It provides a new way to utilize enzymes for biomass pretreatment.

## Anaerobic Digestion

During the anaerobic digestion of biomass, many factors would influence the efficiency. Various efforts have been focused on optimization of the reaction parameters. Li and coworkers demonstrated that the addition of zero valent iron (10 g/L) could hamper the anaerobic digestion of raw biomass. They found that proper amount of zero valent iron would promote the anaerobic digestion activity. An interesting finding of their study was that zero valent iron could largely enhance the methanogenic rate in 6 days, but decreased the total methane yield by 10.3%. By studying varying effects, they got the conclusion that the behavior was impacted by features of substrate and component ratio.

## Biomass Catalytic Pyrolysis

Pyrolysis is one of crucial tools to upgrade biomass into value-added products. Bi and coworker studied the cooperation effects of catalyst component on the pyrolysis process compared with the unitary candidate in the microwave reactor. They investigated the deoxygenation behavior and reaction kinetics including pyrolysis kinetics and pathway of biomass-derived monomers (i.e., cellulose, hemicellulose, and lignin). They found the activation energy of 10KP/10Bento and 10KP/10Clino (the mixture of 10% K_3_PO_4_ plus 10% clinoptilolite) was a little lower or close to those of other candidates at 30 wt.%. They got the conclusion that catalyst mixtures could enhance the catalytic activity clearly, which is attractive to lower the synthesis cost of bio-oils and biochar.

## Degradation of Side Products

Biomass-derived bisphenol A (BPA) is a widely utilized fine chemical in various areas (e.g., medicine and organic synthesis), while the residual BPA is difficult to be degraded by nature. Yan and coworkers reported a photocatalytic route to degrade BPA using a porous ZnO photocatalyst. The effects of various parameters were investigated and porous ZnO photocatalyst can remove 99% BPA in 1 h. The results of EPR analysis confirmed that h^+^, ·O^−2^, and e^−^ played an important role in the removal efficiency. This study offers a photocatalytic route to deal with biomass-derived chemicals.

## Synthesis of Biomass-Derived Chemicals

A variety of biomass-derived chemicals can be selectively produced from biomass, among which cyclopentanol can be used as a versatile eco-friendly solvent in various applications. Zhang and coworkers fabricated a bimetallic Ru-Mo catalyst for hydrogenation-rearrangement reactions of furfurals, achieving 89.1% cyclopentanol yield using 1%Ru-2.5%Mo/CNT pre-reduced at 600°C. The weak acidity and strong hydrogenation activity of the bimetallic Ru-Mo catalyst were found to be important for the synthesis of cyclopentanol from furfural-like compounds.
